# Evolutionary explanations in medical and health profession courses: are you answering your students' "why" questions?

**DOI:** 10.1186/1472-6920-5-16

**Published:** 2005-05-10

**Authors:** Eugene E Harris, Avelin A Malyango

**Affiliations:** 1 Department of Biological Sciences and Geology, Queensborough Community College, City University of New York, New York City, USA; 2 Department of Cell Biology, New York University School of Medicine, New York City, USA

## Abstract

**Background:**

Medical and pre-professional health students ask questions about human health that can be answered in two ways, by giving proximate and evolutionary explanations. Proximate explanations, most common in textbooks and classes, describe the immediate scientifically known biological mechanisms of anatomical characteristics or physiological processes. These explanations are necessary but insufficient. They can be complemented with evolutionary explanations that describe the evolutionary processes and principles that have resulted in human biology we study today. The main goal of the science of Darwinian Medicine is to investigate human disease, disorders, and medical complications from an evolutionary perspective.

**Discussion:**

This paper contrasts the differences between these two types of explanations by describing principles of natural selection that underlie medical questions. Thus, why is human birth complicated? Why does sickle cell anemia exist? Why do we show symptoms like fever, diarrhea, and coughing when we have infection? Why do we suffer from ubiquitous age-related diseases like arteriosclerosis, Alzheimer's and others? Why are chronic diseases like type II diabetes and obesity so prevalent in modern society? Why hasn't natural selection eliminated the genes that cause common genetic diseases like hemochromatosis, cystic fibrosis, Tay sachs, PKU and others?

**Summary:**

In giving students evolutionary explanations professors should underscore principles of natural selection, since these can be generalized for the analysis of many medical questions. From a research perspective, natural selection seems central to leading hypotheses of obesity and type II diabetes and might very well explain the occurrence of certain common genetic diseases like cystic fibrosis, hemochromatosis, Tay sachs, Fragile X syndrome, G6PD and others because of their compensating advantages. Furthermore, armed with evolutionary explanations, health care professionals can bring practical benefits to patients by treating their symptoms of infection more specifically and judiciously. They might also help curtail the evolutionary arms race between pathogens and antibiotic defenses.

## Background

The science of Darwinian or Evolutionary Medicine was formalized in the early 1990s most notably by the eminent evolutionary biologist George C. Williams and the physician and professor Randolph Nesse [1,2]. Its methodologies and aims are directed at the scientific investigation of *evolutionary causes *for human disease, disorders, malfunctions and apparent design failures in order to improve health care and to stimulate fruitful research directions.

To clarify what we mean by evolutionary causes it is useful to make a distinction from a different level of causality in biology, that of proximate causes. Proximate causes are at the level of the immediate mechanisms that give rise to disease, disorders, and malfunctions. These include detailed accounts of physiological processes, embryological development, and anatomical relationships and functions.

Evolutionary explanations, in contrast, are obtained via researchers taking an historical perspective (see Mayr [3]). They look back in time, through lenses of scientifically established evolutionary principles, in order to investigate medical problems and ailments. An evolutionary approach would ask such questions as: "Why hasn't natural selection eliminated a particular disease?" "Why do our bodies have certain flaws of engineering that make us susceptible to particular medical complications?" "Might certain modern diseases and ailments be the result of a mismatch between our biological heritage and our modern-day lifestyles?" and "Are characteristic physiological responses to disease in fact adaptive defenses that have developed over evolutionary time?"

Neither approach, proximate or evolutionary, can be ignored. In fact, such explanations should in theory feedback positively to stimulate novel research approaches [2]. Nonetheless, we realize that evolutionary explanations may seem foreign to many persons in the medical field. After all, most of modern day medicine is based on scientifically discovered proximate explanations, and these explanations dominate in medical textbooks. Thus, to illustrate the evolutionary perspective, in this paper we give a variety of examples for which evolutionary explanations are known or hypothesized. We also describe the theoretical evolutionary principles that underpin these examples and that can be generalized to the investigation of many medical questions.

### Complications of human child-birth: proximate causes

Many of our students are perplexed when they hear about the pain accompanying child delivery. They wonder "Why is there so much pain?" and "Why is the birthing process such a complication, so much so that many doctors deliver by Caesarean section?" The proximate answer describes that the diameter of the human fetus's head is very large (the widest part of the fetus) and the diameter of pelvic outlet of the mother is only slightly bigger. This makes it a very tight passage for the fetus, a very painful process for the mother, and a complicated process for the obstetrician to manage. The release of the hormone relaxin during pregnancy (a proximate mechanism) helps to loosen the pubic symphysis and dilate the uterine cervix easing the complication to a degree. It is easier all around, even if not always necessary, if the fetus can detour the natural route and be delivered through an alternate passageway created in the mother's lower abdomen.

Being accustomed to such explanations, many students appear satisfied, though this is likely a cover up. Curious students naturally want further explanation, "But why is a human infant's head so big?" "Why isn't the mother's birth canal larger? And finally "Why is the human birthing process so complicated? These questions have evolutionary explanations – explanations that may only rarely emerge in medical and health profession's classes.

### Complications of human child-birth: evolutionary causes

Following is a classic evolutionary explanation backed by considerable paleoanthropological evidence. Beginning about 2.5 million years ago, the evolutionary lineage leading to modern humans shows a trend towards increasing brain size so that by around 500,000 years ago our ancestors had brains (and crania) about as large as they are today [4]. However, despite the advantages, evolving such large brains posed serious problems for mothers who need to pass their infant's head through a relatively small pelvic canal [4,5].

"But if brain size evolved to its modern size so long ago, why hasn't evolution had enough time to fix the *imperfect*-fit... that is, why isn't the birth canal larger?" Well, females have indeed evolved a rounder pelvic opening to facilitate the uneasy passage of the newborn's head. This explains the anatomical differences we observe between male and female pelves. There is, however, a limit to the degree to which the female pelvic opening can enlarge. This constraint arises because of another unique human adaptation namely bipedalism – walking on two legs. Exceptional increase of the birth canal would require repositioning of the gluteal pelvic-abductors in such a way as to make bipedalism mechanically and energetically inefficient. Thus, the overall size of the birth canal can be viewed as an evolutionary compromise between two advantageous features of human evolution – increased brain size as well as the new style of locomotion. These two different evolutionary pressures have led to the difficult and *imperfect *process of human child birth [4,5]. (For further discussion of evolutionary obstetrics see Travathan's book *Evolutionary Medicine *[6]).

### The example of sickle cell disease: a proximate perspective

Take as another example, sickle cell anemia. A student asks, "Why does sickle cell disease exist?" The proximate (or mechanistic) explanation is necessary but not sufficient. A point mutation at the sixth amino acid position of the β hemoglobin chain replaces glutamic acid with valine and produces the so-called abnormal hemoglobin type Hb^S ^(the normal type is Hb^A^). When a cell having only the Hb^S ^type gives up oxygen to body tissues it loses the normal red blood cell shape (symmetrical biconcave) and becomes crescent or sickle-shaped. These homozygous individuals (Hb^S^/Hb^S^) develop sickle cell disease in which sickle-shaped RBCs block small peripheral blood vessels leading to very serious secondary effects. Without regular medical intervention few persons live to adulthood [7]. Promisingly, with the recent development of modern treatment regimens (e.g. stem cell transplantations, serial blood transfusions, and prophylactic treatments with penicillin) patients show increased survivability, living into their 40s and 50s and in some cases even older [8,9]. Unfortunately, many children with sickle cell disease do not have access to intensive treatment programs, either because it is not locally available or their health insurance plans limit access to it [10].

Many of our students are older students and have worked in hospitals or clinics in the New York City area. Most are aware that sickle cell disease is commonly encountered. On average, 1 in every 600 births in the African-American population is to a baby with sickle cell disease [11]. Therefore, these students will wonder, "If sickle cell disease is so lethal, then why hasn't it been eliminated?"

### Sickle cell disease: an evolutionary perspective

An evolutionary explanation is that sickle cell anemia is an example of a disease caused by a gene that can also be beneficial. The Hb^S ^type is found at high frequencies (up to 20% and above) in much of tropical Africa. In most other parts of the world the frequency is very low (<1% or absent), excepting for regions such as the Mediterranean, the Arabian Peninsula, and the Americas where a high frequency is due to historic migration and admixture [7]. Importantly, within Africa the distribution of the (Hb^S^) almost exactly matches the distribution of the mosquito-transmitted disease known as malaria, caused by the parasite *Plasmodium falciparum*. It is known that heterozygotes (Hb^S^/Hb^A^), having both types of hemoglobin (and who have only minor health problems), show resistance to malarial infection because the body targets the *P. falciparum *infected cells for destruction. In contrast, individuals homozygous for normal hemoglobin (Hb^A^/Hb^A^) suffer high mortality rates in early childhood due to malarial infection. Thus, the allele for sickle cell has been maintained because heterozygotes have a higher reproductive success than either of the two possible homozygotes. The evolutionary process explaining the phenomenon is known as balancing selection, a special form of natural selection that operates to keep two or more beneficial alleles at relatively high frequencies [7].

This classic example is known since the 1950s [12] and the relationships between mosquito ecology, agriculture, malaria, and frequencies of the sickle cell gene have been carefully studied [13]. Notwithstanding our knowledge, clear explanations of the evolutionary processes that underlie sickle-cell disease, *balancing selection *and *heterozygote advantage*, are still not found in many medical and pre-professional health texts leaving students needlessly wondering 'Why?' Indeed, understanding these evolutionary processes would help students understand why genes underlying other hemotological disorders like alpha and beta thalassemias, Hemoglobin E (Hb E) syndrome, and Glucose-6-phosphate dehydrogenase (G6PD) deficiency are found at high frequencies in certain populations from the Mediterranean, African and/or South East Asia [14,15]. Like with sickle cell, evidence suggests that natural selection has maintained these disease genes because they too confer resistance to malaria [14,15]. While these diseases were once geographically confined, they are now, due to forced, historic, or recent emigration, seen commonly in US or European hospitals and clinics. For example, Glader and Look [16] report that 41% of the South East Asian refugee population in the United States are carriers of, or have, one of the myriad hematological disorders. While in past decades it may have been rare for our students to come into contact with these diseases; today the likelihood is much greater.

#### Explaining symptoms

Evolutionary causes can also explain classic symptoms of illness. Traditionally, we teach that symptoms like fever, inflammation, diarrhea, coughing, vomiting are problems that need to be alleviated by medication. The proximate mechanisms of these symptoms are described in most textbooks. Not sufficiently discussed, though, are evolutionary explanations. These explanations derive from the ongoing evolutionary arms race between pathogens and us, each one trying to out-perform the other. Therefore, to what extent are so-called "symptoms" the body's way of defending itself, and to what extent are they manipulations by the pathogen, "intended" to increase its reproduction and spread?

Studies suggest that fever is an evolved defense mechanism against pathogens that if reduced by medicinal drugs can, in some cases, prolong illness and cause secondary infections [1,2,17]. Diarrhea has been hypothesized to be either a defense mechanism or a manipulation by the pathogen, or even both simultaneously [17]. Persons with *Shigella *bacteria develop severe bloody diarrhea. To test the diarrhea-as-defense hypothesis persons were treated with and without the anti-diarrhea medication Lomotil™. Results showed that illness was prolonged in the treated group as compared with the untreated group [18]. In contrast, *Vibrio cholerae*, the bacterium that causes cholera, carries a toxin on its cell membrane that induces diarrhea, presumably to help its spread. Infected people are severely affected and at risk for death due to loss of body fluids. In this case, treatment with anti-diarrhea medication may counter the pathogens strategy and aid against dehydration and against spread of the bacteria [17]. Ideally, the physician would want to know when defenses are useful to the patient and when they are manipulations by pathogens [1,17]. If each pathogen-host combination were to be analyzed separately, realizing that the balance of evolutionary forces acting on the host and pathogen may vary in each case, then treatment would likely become more specific and effective.

## Discussion

### Evolutionary explanations & natural selection

Can our students benefit from such evolutionary explanations? Yes, we believe they can. In fact, this is the central tenet of the science of Evolutionary Medicine: that principles of evolutionary theory can provide a unifying principle by which human biology, disease, and disorders can be understood [1,2]. The explanatory power lies in fully understanding how natural selection operates.

Following we explain several important principles of natural selection that are generally applicable to understanding human biology in health and disease (Table 1, and see [1,2]).

**Table 1:** Principles of natural selection generally applicable to understanding human biology in health and disease.

**Natural selection**:
• cannot build perfect designs because it compromises between different adaptations – bipedal walking vs. large brain size.
• will maintain a disease gene if it confers an advantage in a particular environment – sickle cell disease, alpha- & beta-thalassemia, Hb E syndrome, G6PD deficiency, Tay-sachs, cystic fibrosis.
• has shaped many human genes to ancient lifestyles (i.e. a hunter-gathering versus modern life-way) explaining chronic diseases like obesity, and type II diabetes.
• favors genes maximizing reproduction even if they compromise health (PKU, hemochromatosis, fragile X syndrome etc.) or longevity (Alzheimer's, atherosclerosis, prostate hyperplasia)
• explains the "arms race" between pathogens and us. We have evolved defenses both natural – fever, diarrhea, vomiting, inflammation – and manufactured – antibiotics. Pathogens evolve counterstrategies like antibiotic resistance, and manipulation of our defenses for their spread.

• *Natural selection cannot build perfect designs because of compromises that exist between different adaptations *(e.g. a pelvic girdle designed for bipedal locomotion versus one designed to facilitate passage of a large brained infant). Almost every human structure and physiological process can be analyzed in terms of compromises between costs and benefits. For example, building bones that will resist fracture would require further increasing their thickness and/or calcium content, though doing so would make us slower and take away calcium crucial for normal cellular functions. Indeed, a homeostatic mechanism has evolved that draws calcium from bones when cellular functions need it. However, that same mechanism can sometimes overdraw the mineral producing problems such as fracture, osteomalacia, and rickets.

• *Natural selection works within the limitations imposed by the human body's long evolutionary legacy*. George C. Williams has rhetorically questioned: why hasn't the birth canal evolved to open through the lower abdomen rather than through the pelvis, thus releasing the severe evolutionary constraint against further increase in brain size? The answer is simply that vertebrates set upon routing the female reproductive tract through the pelvic opening hundreds of millions of years ago and there has been no way for evolutionary processes to readjust the route [19].

• *Natural selection may often adapt our genes to the context of a particular environment*. Therefore, certain genotypic traits offer benefits only with respect to the environment in which they evolved (e.g. genes causing sickle cell disease and other hemoglobinopathies). When these genotypes are expressed in other environments, like sickle cell is in the United States, there is essentially no benefit because malaria is largely absent. As expected, the frequency of the sickle cell gene is declining among American Blacks [7]. As another example, in 1987 Rotter and Diamond [20] hypothesized that the high frequency of the genes causing Tay sachs (homozygote frequency of 1 in 6000 births) and cystic fibrosis (homozygote frequency of 1 in 1700 births), in individuals descended from Ashkenazi Jews and European populations respectively, results from the survival advantage the genes conferred to heterozygote individuals who endured past epidemics of tuberculosis and/or typhus.

Such evolutionary hypotheses can be tested scientifically, for example, witness studies of cystic fibrosis (CFTR) [21,22]. The gene was recently found to code for chloride ion protein-channels on somatic epithelial cell membranes, and the bacteria causing typhoid fever, *Salmonella enterica *serovar Typhi, was found to target the CFTR protein in GI cells to gain entry to them. Subsequent tests showed that serovar Typhi invasion rates in mice are reduced by over 80% in heterozygotes for the mutant allele (ΔF508 CFTR) compared to homozygotes (for the normal CFTR allele) due to reduced CFTR expressed on the cell surface. Moreover, when European populations were analyzed that reportedly suffered Typhoid scourges in the past, it was found that they showed higher frequencies for the mutated allele one to two generations after the scourge [22]. Together, the proximate and historical evidence strongly support Typhoid fever as an important selective agent (though perhaps not the only agent) that drove cystic fibrosis frequencies upwards due to heterozygote advantage. In contrast, it is unfortunate that the Tay sachs selective hypothesis remains largely untested almost two decades later.

• As an extension of the previous principle, *natural selection has likely optimized most of our genes to the environment and lifestyle by which we have lived the longest – a hunter-gatherer lifestyle *– likely explaining the presence of certain chronic diseases (type II diabetes, obesity and others). Our genes and homeostatic mechanisms evolved under vastly different environmental and nutritional conditions than those under which we live today [23-25]. For example, with respect to obesity, much recent research has focused on leptin, a hormone released by adipose tissue that regulates appetite by acting on the hypothalamus. Although further research is required, evidence suggests that under increasingly obese body conditions the brain becomes resistant to leptin throwing the leptin-hypothalamic homeostatic system into an unhealthy positive feedback loop that further stimulates appetite [26,27]. With respect to modern diets and lifestyles – high in calories and low in physical activity – the advantage of such a system makes little sense. However, under presumed ancient lifestyles, in which caloric intake almost balanced caloric consumption, the advantage of such a "thrifty genotype" becomes clearer especially if it helped our ancestors survive intermittent episodes when food was sparse [26-28].

• *Natural selection operates to maximize the number and survival of one's offspring (and thereby one's genes) in the next generation and not to maximize health or longevity*. Regarding longevity, dramatic examples of this principle are seen in many insects in which aging and death is swift or almost immediate after mating. In humans, it seems to account for the process of human senescence – the general deterioration of the body with age and the rather sharp up-turn in chronic diseases in post-reproductive years. Natural selection selects genes on the basis of the benefits they provide early in life even if they may cause adverse effects later on, a concept known as *antagonistic pleiotropy *[1,29](i.e. a gene having multiple but antagonistic effects). For example, an efficient mechanism of calcium deposition evolved to repair bone fractures early in life may lead to arteriosclerosis, excessive calcium build-up in arterial walls, later in life [1,29]. Unfortunately, the concept of antagonistic pleiotropy (and this possible example of it) advanced by George C. Williams in 1957 [29], has never been rigorously tested. Nonetheless, the concept has been recently applied in attempts to explain Alzheimer's disease, prostate hyperplasia, and certain types of cancer [30]. Regarding Alzheimer's disease, a recently advanced evolutionary hypothesis [31,32] is built on findings of neuroprotective functions for estrogen and apolipoproteins E2 and E3. As estrogen declines in post-menopausal women, these apolipoproteins (uniquely evolved in humans) help delay the onset of Alzheimer's-like pathologies (and interestingly cardiovascular disease). With longevity increases over time in the human evolutionary lineage, older women having the neuroprotective apolipoproteins would have enjoyed a selective advantage since their ability to competently care for their grandchildren (with whom they share 1/4 of their DNA) was prolonged [31,32]. This special form of natural selection, in which a person gains a benefit by helping close relatives, is termed *kin selection *[1,33]. This hypothesis of the evolution of Alzheimer's has generated much commentary and many scientifically testable predictions (see [31] and associated commentaries).

Furthermore, the principle that natural selection promotes reproductive success and not necessarily health seems to explain the maintenance of certain common disease genes because of possible compensating advantages. Following are several examples. Homozygotes for phenylketonuria (PKU; frequency of 1 in 10,000 Caucasian births) develop mental retardation because they cannot metabolize the amino acid phenylalanine, but heterozygote infants seem to show decreased probability of being miscarried. Hemochromatosis is a very common autosomal recessive disease in individuals of Northern European descent (homozygote frequency of 1 in 200 births) characterized by increased intestinal iron absorption. It has been hypothesized that the gene may be advantageous to women by compensating for high iron losses (through menstruation, pregnancy, and milk production.) [20] On the other hand, men with the gene may suffer from excessive iron stores late in life. Supporting evidence may be seen in the recent finding of increased longevity of female carriers over non-carriers [34] and recent population genetic analyses of SNPs that have revealed positive natural selection in the gene's history [35]. More recently, a different hypothesis has been advanced based on the finding that individuals bearing the hemochromatosis mutation (C282Y) show partial resistance to *Yersinia*, the bacterium causing plague [36]. In normal individuals, *Yersinia *initially multiply within iron-rich macrophages, however this does not occur in persons with C282Y because their macrophages lack iron, a mineral essential for the pathogen's survival. This evolutionary hypothesis, which requires further testing, suggests that natural selection favored persons bearing the C282Y allele during the devastating plague that ravaged Europe during the Middle-ages [36].

Compensating advantages may also maintain disease genes associated with diabetes Type I (reduced miscarriage when linked to the beneficial HLA DR3 gene [1,20]), Fragile X syndrome (increased fertility among heterozygous females [37] or prenatal advantages to offspring [38]), gout and hyperuricemia (beneficial antioxidant effects [39,40]) among other examples [1,2,6,20]. Surprisingly, many such evolutionary hypotheses initially advanced a decade or more ago on the bases of observed biased inheritance patterns, or because of the commonness of the disorder and other circumstantial evidence, have not received rigorous scientific testing. In fact, for each disease or disorder, predictions could be made with respect to its association with a hypothesized compensating advantage, and experiments designed to test the predictions. It would then be possible to determine the validity of the evolutionary hypotheses, and to clarify the proximate genetic and physiological mechanisms involved.

## Summary

We have described different aspects of human biology of medical significance. All have evolutionary explanations rooted in evolutionary theory. There are, of course, many more such explanations that can be incorporated into our texts and into medical curricula [1,2,6]. We recommend Nesse and William's thought-provoking book on this subject as a good and comprehensive introduction to the field [1]. Indeed, summaries of topics exist that could serve as syllabi for medical courses [41-43]. If we can help answer our student's questions by providing them with evolutionary answers (to the extent possible given the field's current knowledge) and by sufficiently describing evolutionary principles, we will do them a large favor. They will gain a powerful set of organizing principles around which they can arrange the enormous amounts of proximate information they must learn.

Furthermore, those of our students who in the future will do basic and clinical research may indeed find it fruitful to apply evolutionary theory and methods in order to understand problems of medical interest. Evolutionary medicine can have enormous potential. For example, one recent evolutionary analysis identified genes that in the past seem to have enabled viruses in the smallpox and vaccinia family to evade our defenses, genes that could be targeted for drug design today [44]. Furthermore, as described above leading hypotheses of the origins of type II diabetes [27,45], and obesity [26,27] have evolutionary explanations at their cores.

As a parting thought, imagine that the questions our students ask are like the endless series of "why" questions children ask, that go on (and on and on)! Such questions are also like their answers. They cycle on in a positive feedback loop yielding greater illumination. Although it is crucial we study the proximate mechanisms of disease, evolutionary theory offers a scientific methodology that can lead us to examine these mechanisms in novel ways. If we do not give our students support in asking such questions, might they stop asking them? What would be the consequences of this? Finally, as food for thought, we ask you to consider the following question: are *you *answering your students' "why" questions?

## Competing interests

The author(s) declare that they have no competing interests.

## Authors' contributions

Both authors contributed equally to this work.

## Pre-publication history

The pre-publication history for this paper can be accessed here:


